# A missense variant in the *SOX5* gene (c.221C > T) is associated with intellectual disability

**DOI:** 10.1186/s13023-025-03548-z

**Published:** 2025-02-04

**Authors:** Xiujuan Yang, Zhongzhi Gan, Xiaoling Guo, Xiang Huang, Juan Liu, Yingchun Zheng, Xiaoqiang Zhou, Jingli Lian, Yue Liu, Tingting Yang, Chao Li, Fenying Chen, Fei He, Xiangmin Xu, Yasi Zhou, Qian Liu, Xingkun Yang, Fu Xiong

**Affiliations:** 1 The Affiliated Foshan Women and Children Hospital, Guangdong Medical University, Foshan, 528000 China; 2 Department of Medical Genetics/Experimental Education/Administration Center, School of Basic Medical Sciences, Southern Medical University, Guangzhou, 510515 China; 3 Clinical Research Center of Scientific Research Division, Nanfang Hospital, Southern Medical University, Guangzhou, 510515 Guangdong China; 4 Guangdong Provincial Key Laboratory of Single Cell Technology and Application, Guangzhou, 510515 China; 5 Department of Fetal Medicine and Prenatal Diagnosis, Zhujiang Hospital, Southern Medical University, Guangzhou, 510280 China; 6 The First Clinical Medical School, Nan Fang Hospital, Southern Medical University, Guangzhou, China; 7 Foshan Pulisheng Biotechnology, Foshan, 528000 China

**Keywords:** Intellectual disability, *SOX5*, C.221C > T, Bioinformatics prediction, Protein stability, Down-regulate

## Abstract

**Objectives:**

The *SOX5* gene has been identified as the pathogenic gene responsible for Lamb-Shaffer syndrome. In this study, we examined the *SOX5* variant (c.221C > T, p.Thr74Met) within a Chinese family presenting with intellectual disability and evaluated the functional implications of *SOX5* by in vitro experiment.

**Materials and methods:**

The family underwent a clinical assessment of intellectual development, which included precise clinical exome sequencing to identify causative genetic variants. The potential deleterious effects and pathogenicity of the variant were predicted using bioinformatics tools such as Mutation Taster, PROVEAN, and SIFT. Additionally, protein stability was evaluated using I-Mutant, and 3D protein structures were modeled with I-TASSER. Western blots and QPCR were employed to assess gene expression and protein stability. Flow cytometry was utilized to compare the cell cycle dynamics between wild-type and mutant cells.

**Results:**

A previously identified missense variant (c.221C > T) in the *SOX5* gene was determined to be the underlying cause of intellectual disability in a Chinese family. Functional assays demonstrated that mutant cells exhibited increased levels of *SOX5* mRNA and protein relative to wild-type cells, accompanied by enhanced protein stability. Additionally, the mutant *SOX5* protein was found to alter the cell cycle and downregulate the mRNA expression levels of the *ACAN*, *AXIN2*, *SOX9*, and *PDGFRA* genes.

**Conclusions:**

We confirmed that the *SOX5* p.Thr74Met variant is associated with intellectual disability in a second-generation Chinese family. This mutant protein potentially exhibits increased stability, influences the cell cycle, and downregulates genes related to bone and neural functions.

## Introduction

The *SOX5* gene, located on chromosome 12p12.1, encodes a transcription factor for the regulation of cell fate and differentiation during neurogenesis, bone development and the development of other tissues. In the context of tumors or inflammatory conditions, the *SOX5* protein predominantly participates in processes such as cell proliferation, apoptosis, cell cycle regulation, inflammatory response, as well as cellular migration and invasion [1–3]. *SOX5*, *SOX9* and *SOX6* constitute the SOX trio, collaboratively facilitating the activation of target gene expression, including Col2a1 and Acan, during the process of cartilage differentiation [4]. Furthermore, *SOX5* influences bone differentiation and neural development. In conjunction with *SOX6*, *SOX5* modulates neural stem cell proliferation, neuronal diversity, neuronal migration, and projection formation in vertebrates [5, 6]. Additionally, *SOX5* is instrumental in promoting the cell cycle exit of neural progenitor cells, and its downregulation is essential for the progression of neuronal differentiation [7, 8]. *SOX5* serves as a critical regulator of gene expression profiles during neurogenesis within the cortex, spinal cord, and neural crest. In the cortical region, *SOX5* orchestrates the sequential production of distinct corticofugal neuron subtypes by inhibiting the inappropriate and premature emergence of corticofugal neurons that typically develop later [9]. Within the spinal cord and telencephalon, the deficiency of *SOX5/SOX6* in oligodendroglia can result in premature differentiation, consequently impairing the migratory capacity of oligodendrocyte progenitor cells [10]. In the neural crest, it has been demonstrated that *SOX5* regulates the formation of neural crest cells and influences the rate of neurogenesis in chick embryos [6].

Lamb-Shaffer syndrome (OMIM 616803) is attributed to variants in the *SOX5* gene, following an autosomal dominant inheritance pattern. This syndrome is clinically characterized by intellectual disability, language delay, dysmorphic features, behavioral deficits and motor dysfunction [7]. Haploinsufficiency of the *SOX5* gene results in neurodevelopmental delays, potentially leading to a spectrum of developmental disabilities ranging from mild to severe, as well as language impairments and dysmorphic characteristics [11, 12]. Furthermore, a variant in the *SOX5* gene alters cartilage and growth plate differentiation, leading to decreased height and mild skeletal dysplasia [13]. Presently, the majority of intellectual disabilities and bone developmental disorders associated with *SOX5* are dominant disorders resulting from haploinsufficiency [14]. Nevertheless, the presence of both common and distinct manifestations suggests a heterogeneous clinical phenotype within the same genotype. For instance, five patients have been documented with heterozygous variants: (c.1672C > T, p.Arg558Trp) and (c.1711C > T, p.Arg571Trp) [15]. Among the five patients studied, three exhibited hypotonia, whereas only one showed signs of microcephaly. Additionally, scoliosis was observed in all three cases with the *SOX5* variant (c.1477C > T, p.Arg493*); however, dysmorphic facial features and abnormalities in the hands or feet were present in only one of these cases [16].

This study reports on a Chinese family with a *SOX5* variant associated with intellectual disability. Notably, unlike previous cases where *SOX5* haploinsufficiency was implicated in the disease, this particular *SOX5* variant (c.221C > T, p.Thr74Met) demonstrates a dominant negative effect.

## Materials and methods

### Subjects

A normal adult male (II:1) presented to our hospital seeking genetic counseling, prompted by the presence of similar diseases in two family members (I:1 and II:2). Peripheral blood samples were collected from all participants, including two affected individuals (I:1 and II:2) and two unaffected individuals (I:2 and II:1). Comprehensive clinical examinations and high precision clinical exon sequencing were conducted at the Foshan Women and Children Hospital. The genetic analysis identified a variant in the *SOX5* gene.

### Variant analysis

Genomic DNA was extracted according to the standard operating instructions of the nucleic acid extraction or purification kit (AmCare Genomics Lab, Guangzhou, China). Then the DNA was digested and fragments were modified by adding an “A” base at the 3 ‘end and ligated to the adaptors. Custom-designed Amcare probes (AmCare Genomics Lab, Guangzhou, China) were used for in-solution hybridization to enrich target sequences, which included coding exons for about 5000 clinically relevant disease-causing genes. The genes were selected based on reports in OMIM, HGMD, and peer-reviewed literature. Known pathogenic variants in deep intronic and other non-coding regions in targeted genes were also included. The libraries of genomic DNA samples were prepared using the Gene Sequencing Library Kit (Random Endonuclease Method) (AmCare Genomics Lab, Guangzhou, China), then adaptors were added and they were amplified with pre-capture ligation-mediated PCR (LM-PCR). The quality and fragment size of the DNA samples in the library were assessed using 1.0% agarose gel electrophoresis. The multifunctional microplates (Molecular Devices) were used to detect the concentration of the library. Enriched DNA samples were indexed and sequenced on the AmCareSeq-2000 sequencer (AmCare Genomics Lab, Guangzhou, China). The average coverage depth was 200 × with > 98% of the target regions covered by at least 20 reads.

### Bioinformatics

The raw reads in fastq format were filtered for adapter sequences, polyN, polyA, and other sequences using the fastp software, and low-quality reads (QC less than 20) were removed to obtain clean reads for subsequent data analysis. The sequencing reads were then mapped to the reference human genome version hg19 using Burrows-Wheeler Aligner (BWA, v0.7.15). Harmful and pathogenic variants were predicted by Mutation Taster (https://www.mutationtaster.org/), PROVEAN and SIFT (http://provean.jcvi.org/protein_batch_submit.php?species=human). The protein stability was predicted by I-Mutant (https://folding.biofold.org/i-mutant/i-mutant2.0.html). The three-dimensional (3D) protein structures of wild-type and mutant *SOX5* proteins were predicted using I-TASSER (https://zhanggroup.org//I-TASSER/). The sequences of several species were compared to the reference sequence from the NCBI database (https://www.ncbi.nlm.nih.gov/homologene/) to understand the conservation of the variant site across different species.

### *SOX5* expression plasmid constructs and mutagenesis

Total RNA was isolated from K562 cells using RNAex Pro Reagent (Accurate Biology, Hunan, China). The first-strand cDNA was then synthesized using the HiScript II 1st Strand cDNA Synthesis Kit (Vazyme, Jiangsu, China). The *SOX5* coding sequence was amplified using the Table 1 primers, designed with EcoRI and BamHI restriction sites. After double digestion with EcoRI and BamHI (New England Biolabs, Beijing, China), the PCR products were cloned into the pEGFP-N1 vector. The c.211C > T variant was amplified by PCR site-directed mutagenesis, using wild-type plasmid as template. The site-directed mutagenic primers were designed as shown in Table 1. Recombinant plasmids were purified using the EndoFree Mini Plasmid Kit II (TIANGEN, Beijing, China), followed by Sanger sequencing to identify the wild-type and mutant.

**Table 1:** Sequences of the primers used for PCR

Primers	Sequences (5′ to 3′)
*SOX5* coding sequence	Forward primer: 5'-CGGAATTCATGCTTACTGACCCTGATTTACCT-3' Reverse primer: 5'-CGGGATCCCGGTTGGCTTGTCCTGCAAT-3'
*SOX5* mutagenic primer	Forward primer: 5'-AGTTTCTCTGCTGATGCAAGAGACTTGTG-3' Reverse primer: 5'-CACAAGTCTCTTGCATCAGCAGAGAAACT-3'
*SOX5-* qPCR	Forward primer: 5′-GGACTCCCACTTCTCAGCAC-3′ Reverse primer: 5′-TCTGCCTTCTGAGGTGAGGT-3′
*ACAN-* qPCR	Forward primer: 5′-GCCAGCACCACCAATGTAAG-3′ Reverse primer: 5′-TTCAGTAACACCCTCCACGA-3′
*AXIN2-* qPCR	Forward primer: 5′-AGCCAAAGCGATCTACAAAAGG-3′ Reverse primer: 5′-AAGTCAAAAACATCTGGTAGGCA-3′
*SOX9-* qPCR	Forward primer: 5′-AGCGAACGCACATCAAGAC-3′ Reverse primer: 5′-CTGTAGGCGATCTGTTGGGG-3′
*PDGFRA-* qPCR	Forward primer: 5′-TTGAAGGCAGGCACATTTACA-3′ Reverse primer: 5′-GCGACAAGGTATAATGGCAGAAT-3′
*GAPDH-* qPCR	Forward primer: 5′-GTGAAGGTCGGAGTCAACG-3′ Reverse primer: 5′-TGAGGTCAATGAAGGGGTC-3′

### RNA analysis

Human embryonic kidney (HEK) 293T cells and Neuro-2a cells, at 70% confluence, were transfected with 1 μg of the recombinant plasmids containing wild-type (*pEGFP-N1-SOX5-WT*) or mutant *SOX5* c.221C > T genes (*pEGFP-N1-SOX5-MUT*), using Lipofectamine™ 3000 transfection reagent (Invitrogen, NY, USA). After 24 h of transfection, total RNA was isolated using the RNAex Pro Reagent (Accurate Biology, Hunan, China) and reverse transcribed into cDNA using the HiScript III RT SuperMix for Quantitative Real-time PCR (qPCR)(+ gDNA wiper) (Vazyme, Nanjing, China). The relative mRNA levels of wild-type and mutant *SOX5* were then measured using the ChamQ SYBR qPCR Master Mix (Vazyme, Nanjing, China). We overexpressed wild-type and mutant *SOX5* and then tested the expression levels of the *ACAN*, *AXIN2*, *SOX9* and *PDGFRA* genes. The *GAPDH* gene was used as the reference gene to normalize expression. These qPCR primers were shown in Table 1. The 2-ΔΔCt method was used to calculate the levels of gene expression. Transfection and real-time PCR assays were repeated three times to confirm the reproducibility of the results.

### Western blotting analysis and protein stability

To analyze the difference in protein levels between the wild-type and mutant *SOX5* cells, Neuro-2a cells and 293T cells were transfected with 2.5 μg of pEGFP-N1, *pEGFP-N1-SOX5-WT* or *pEGFP-N1-SOX5-MUT* vector using Lipofectamine™ 3000 transfection reagent. Forty-eight hours after transfection, cells were collected, washed with cold PBS (Sigma-Aldrich) and lysed with cell lysis buffer (Beyotime Biotechnology, Shanghai, China) that contained 1% phenylmethanesulfonyl fluoride (Beyotime Biotechnology). After shaking for 30 min at 4 °C, cell debris was removed by centrifugation. We added 5 × sodium dodecyl sulfate–polyacrylamide gel electrophoresis (SDS-PAGE) loading buffer (Beyotime Biotechnology, Shanghai, China) to the cell lysates before boiling for 5–10 min. Protein samples were then separated by a 10% SDS–polyacrylamide gel and transferred onto a polyvinylidene fluoride membrane (Millipore, MA, USA). After being blocked for one hour with 5% skimmed milk at room temperature, the membranes were incubated overnight at 4 °C with green fluorescent protein (GFP)-tagged mouse monoclonal antibodies (proteintech, Rosemont, USA), Cyclin A polyclonal antibodies (Bioworld, Minnesota, USA) or HRP *GAPDH* mouse monoclonal antibodies (proteintech, Rosemont, USA). The following day, membranes were incubated with goat anti-mouse IgG or goat anti-Rabbit IgG (proteintech, Rosemont, USA) at room temperature for 1.5 h after three washes with Tris-Buffered Saline-Tween 20. The immunoreactive proteins were visualized with SuperSignal West Pico ECL (Thermo Fisher Scientific) and a digital chemiluminescence system (Tanon Science & Technology, Shanghai, China). Additionally, to study *SOX5* protein degradation, at 48 h post-transfection, 100 µg/ml of cycloheximide (Sigma-Aldrich) was added to each well. Total cell protein was collected at 0, 2, 4, 6, and 8 h to evaluate the half-life of the *SOX5* protein. Western blot was used to analyze and evaluate the half-life of *SOX5* expression. The protein levels were quantified using ImageJ software. Protein analysis was repeated three times for each test and *SOX5* and Cyclin A levels were normalized to *GAPDH*.

### Subcellular localization

The HEK 293T cells and Neuro-2a cells were cultured in DMEM (Gibco, NY, USA) with 10% fetal bovine serum (Gibco, NY, USA) at 37 °C and under 5% CO_2_. The pEGFP-N1-*SOX5*-WT and pEGFP-N1-*SOX5*-MUT vectors were transfected into the HEK 293T and Neuro-2a cells using Lipofectamine 3000 transfection reagent. After transfection for 36 h, cells were washed three times with PBS and fixed for 30 min with 4% paraformaldehyde at room temperature. After fixation, cells were washed three times with PBS and incubated in 0.2% Triton X-100 (Thermo Fisher Scientific, MA, USA) to increase the permeability of the cytomembrane. Finally, the nuclei were stained with DAPI (Beyotime Biotechnology, Shanghai, China). An inverted confocal fluorescence microscope (LSM 880; Carl Zeiss AG, Jena, Germany) was used for imaging the cells.

### Cell cycle flow cytometry and CCK-8 cell proliferation assay

The pEGFP-N1-*SOX5*-WT and pEGFP-N1-*SOX5*-MUT vectors were transfected into Neuro-2a cells using Lipofectamine 3000 transfection reagent. After transfection for 36 h, the cells were collected using trypsin digestion and perfect medium termination. Cells were washed with PBS and fixed overnight with 70% ethanol at 4 °C and then washed again with PBS. They were then incubated with propidium iodide staining solution (Beyotime Biotechnology, Shanghai, China) at 37 °C in a dark environment for 30 min. After staining, cells were stored at 4 °C or in an ice bath away from light. Red fluorescence and light scattering were detected by flow cytometry at an excitation wavelength of 488 nm. The Flowjo software was used for cell DNA content analysis and light scattering analysis.

The CCK-8 assay kit was used to assess cell proliferation. Neuro-2a cells were first transfected with the *pEGFP-N1-SOX5-WT* and *pEGFP-N1-SOX5-MUT* vectors using Lipofectamine 3000 transfection reagent. The transfection system was set up in a 12-well plate, divided into three groups: the first group was transfected with 1 μg of WT per well, the second group with 1 μg of MUT per well, and the third group was co-transfected with 500 ηg each of WT and MUT per well, for a total of 1 μg. After 24 h of transfection, 10^4 cells per well were seeded evenly into a 96-well plate and cultured for 12, 24, and 36 h. Subsequently, 100 μl of complete medium containing 10 μl of CCK-8 solution was added to each well, followed by incubation at 37 °C for 1.5 h. Absorbance at 450 nm was then measured using a microplate reader.

### Statistical analyses

GraphPad Prism software was used for statistical analyses. The independent samples T test and two-way ANOVA were used to determine the statistical significance between two groups. Statistical data were expressed as the mean ± standard deviation (SD). A *P* value < 0.05 was considered to be significant. *p < 0.05, **p < 0.01, ***p < 0.001 and ****p < 0.0001.

## Results

### Clinical phenotype

**Fig. 1:**
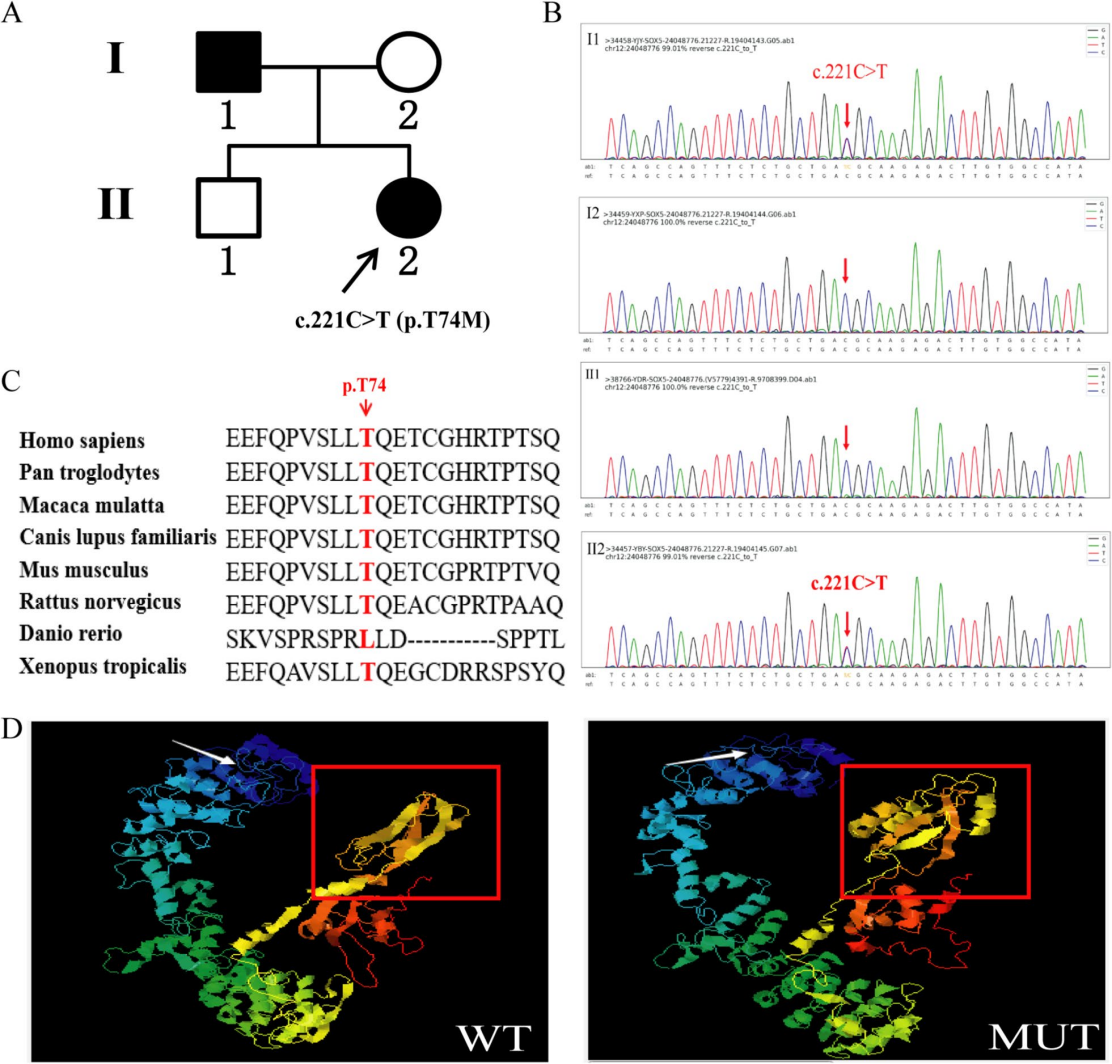
Variant screening and prediction analysis. **A** Pedigree of the family. Males are marked as squares and females as circles. An arrow points out the proband, while the black symbols indicate the affected family member. **B** Sanger sequencing results. All affected members carry the heterozygous missense variant, c.221C > T (p.Thr74Met) in *SOX5*. **C** The amino acid (p.Thr74Met) at this site is highly conserved across numerous species. **D** Protein structure predicted by I-TASSER: wild-type (left, WT) and mutant (right, MUT). A white arrow indicates the site amino acid position 74. The red box represents the location of the HMG domain, which has significant structural changes in mutant type

Clinical evaluations indicated that the proband (II:2), a 30-year-old female, exhibited delayed intellectual development, acquiring the ability to walk at three years of age and developing speech and recognition skills at the age of four. At the age of seven, the proband underwent evaluation using the Wechsler Intelligence Scale for Children, obtaining a score of 52. This scale assesses various cognitive abilities. The proband exhibits normal motor skills, demonstrating the ability to perform self-care tasks, complete simple household chores, and engage in basic communication with family members. The proband's height is 155 cm, and no apparent physical deformities or other abnormal behaviors have been observed. The proband's father (I:1) presented with analogous symptoms, whereas her mother (I:2) and brother (II:1) did not exhibit any related clinical manifestations. The family comprises four members, two affected individuals (I:1 and II:2), and two unaffected individuals (I:2 and II:1). The family pedigree is presented in Fig. 1A.

### Variant analysis

To identify the pathogenic variant responsible for the disease, we performed high precision clinical exon sequencing using genomic DNA from the proband and her parents (II:2, I:1 and I:2). Sanger sequencing was performed on the proband's brother (II:1). The analysis of subject II:2 identified a variation in the *SOX11* gene, NM_003108.4(*SOX11*): c.674A > G (p.Asp225Gly), and a variation in the *SOX5* gene, NM_006940.6(*SOX5*): c.221C > T (p.Thr74Met). According to variant classification guidelines of the American College of Medical Genetics and Genomics (ACMG), the two gene variants are classified as category 3, indicating variants of uncertain significance. These variants were inherited from the father (I:1) and were heterozygous. Additionally, copy number and single nucleotide polymorphism (SNP) analyses did not reveal any copy number variations associated with clinical manifestations. Following Sanger sequencing and co-segregation analyses, it was found that the unaffected proband's brother (II:1) possesses the heterozygous *SOX11* variant (c.674A > G, p.Asp225Gly) and does not carry *SOX5* variants. Additionally, the unaffected family member I:2 does not harbor variants in either *SOX11* or *SOX5*. The sequencing results for *SOX5* among the family members are presented in Fig. 1B. We conducted an analysis of the clinical phenotypes and the functions of pathogenic variants within this family to investigate the pathogenesis associated with *SOX5*.

### Bioinformatics analysis

According to the prediction from Mutation Taster, the c.221C > T (p.Thr74Met) variant in the *SOX5* gene is identified as a disease-causing variant, potentially operating through three pathogenic mechanisms: (1) alteration of the amino acid sequence, (2) modification of protein characteristics, (3) changes in splice sites (Table 2). While PROVEAN software classified the variant as neutral, SIFT analysis indicated it as damaging (Table 2). Additionally, I-Mutant predicted an increase in the stability of the *SOX5* protein (Table 2). Multi sequence alignments of different species from the NCBI database revealed that the amino acid at this site is highly conserved across numerous species, including pan troglodytes, macaca mulatta, canis lupus, mouse, etc. (Fig. 1C). Analysis using the I-TASSER software indicated that the *SOX5* variant changed the tertiary structure of the protein, resulting in the loss of segments of the alpha-helix and random coil structures as highlighted by the red box in Fig. 1D.

**Table 2:** Prediction of harmful pathogenicity

Software	Score	Prediction
Mutation taster	0.9997	Disease causing
Provean	− 0.99	Neutral
SIFT	0.042	Damaging
VarSite (CADD)	22.50	Possibly deleterious
I-Mutant	–	Stability increase

### Functional analysis

**Fig. 2:**
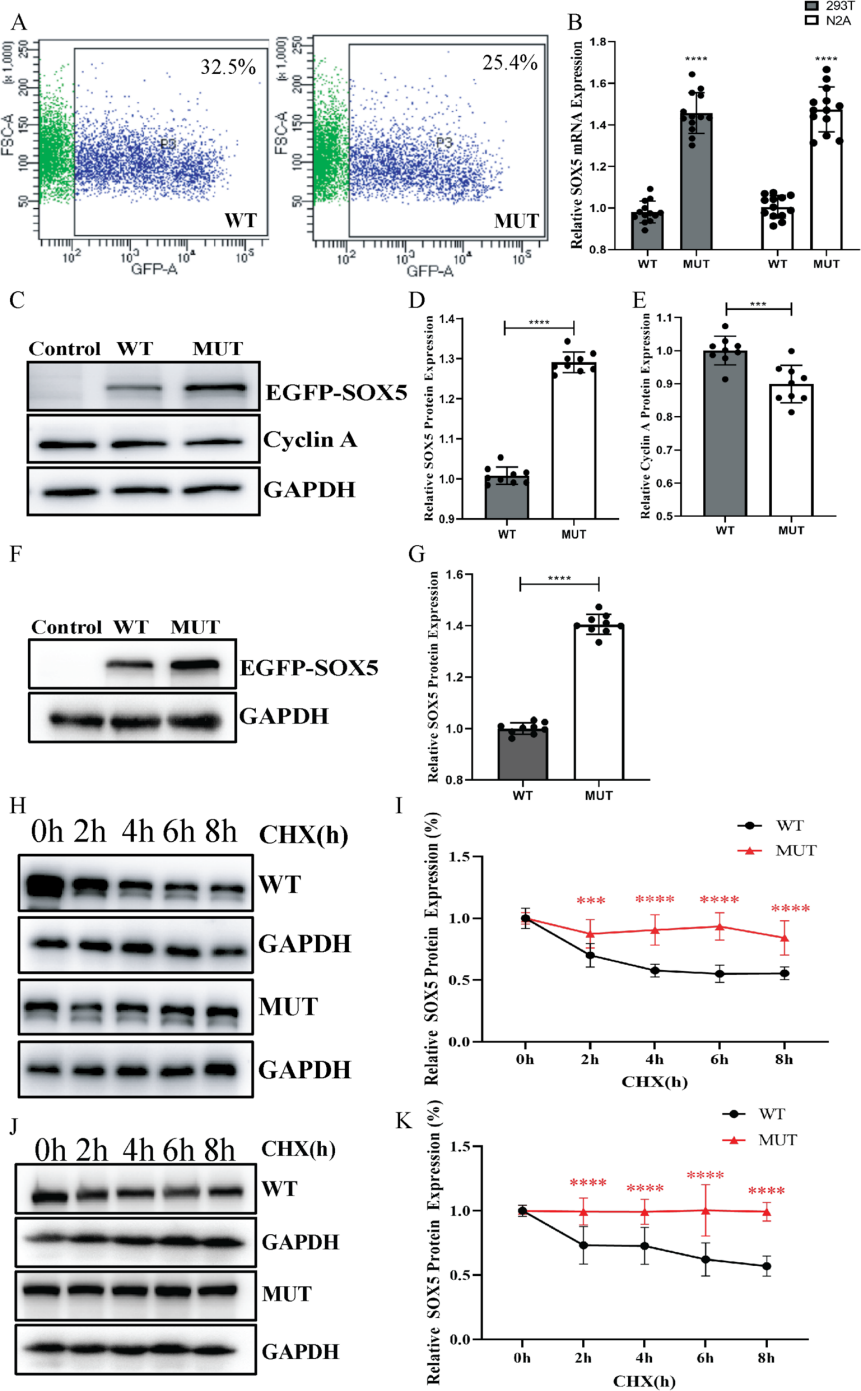
The *SOX5* expression and protein stability analysis. **A** The transfection in Neuro-2a cells was assessed using flow cytometry. The wild-type group exhibited a transfection efficiency of 32.5%, which was higher than the 24.4% observed in the mutant group, and the difference was not significant. Cells expressing GFP are shown in blue, while those lacking GFP are depicted in green. **B** The mRNA expression levels of *SOX5* in 293T cells and Neuro-2a cells. There was a significant increase in the variant mRNA compared with wild-type (****p < 0.0001). **C**–**E** Western blot analysis was conducted to evaluate the expression of *SOX5* and cyclin A in Neuro-2a cells. The mutant *SOX5* exhibited elevated expression levels compared to the wild-type in Neuro-2a cells (****p < 0.0001). The cyclin A expression in the mutant *SOX5* was expressed at a lower level than in the wild-type in Neuro-2a cells (***p < 0.001). **F**–**G** Western blot analysis of *SOX5* in 293T cells. The *SOX5* p.Thr74Met variant was expressed at a higher level than the wild-type in 293T cells (****p < 0.0001). **H**–**I** Western blot analysis of the *SOX5* protein stability in Neuro-2a cells. The *SOX5* protein stability was also increased compared with the wild-type protein in Neuro-2a cells (***p < 0.001, ****p < 0.0001). **J**–**K** Western blot analysis of the *SOX5* protein stability in 293T cells. The *SOX5* protein stability was also increased compared with the wild-type protein in 293T cells (****p < 0.0001)

**Fig. 3:**
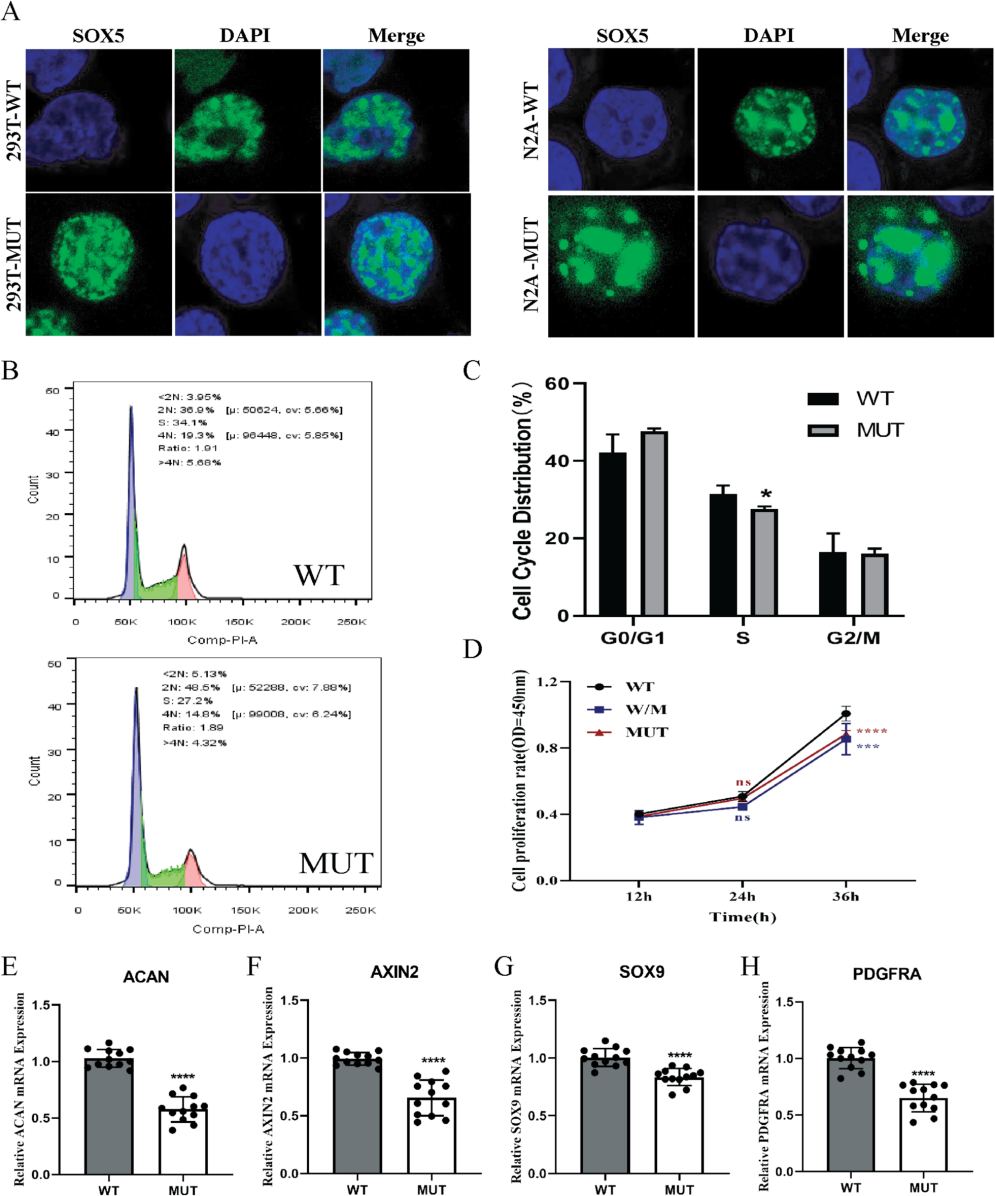
Cell cycle analysis and expression analysis of *SOX5* target gene. **A** Localization of wild and mutant in 293T cells and Neuro-2a cells. Wild-type and mutant *SOX5* were mainly localized in the nucleus in 293T cells and Neuro-2a cells. Confocal images of EGFP (green), DAPI nuclear staining (blue), and merged signals. **B**–**C** Flow cytometry analysis of the cell cycle in Neuro-2a cells: wild-type and mutant. The numbers of S phases in mutant *SOX5* were expressed at a lower level than in the wild-type (*p < 0.05). **D** After transfection of Neuro-2a cells, cell proliferation was detected by CCK-8 assay. **E**–**H** The mRNA expression analysis of *SOX5* target gene in 293T cells. The mRNA expression levels of *ACAN*, *AXIN2*, *SOX9* and *PDGFRA* were decreased compared with the wild-type (****p < 0.0001)

In the Fig. 2, "Control" denotes cells transfected with the empty pEGFP-N1 vector, "WT" indicates cells transfected with *pEGFP-N1-SOX5-WT*, and "MUT" refers to cells transfected with *pEGFP-N1-SOX5-MUT*. The functional effects of *SOX5* variants were evaluated in HEK 293T cells and Neuro-2a cells. The transfection efficiencies of the wild-type and mutant recombinant plasmids were relatively consistent in Neuro-2a cells, with efficiencies of 32.5% for the wild-type and 25.4% for the mutant (Fig. 2A). As illustrated in Fig. 2B–G, significant differences were observed in the mRNA and protein levels between cells transfected with the *pEGFP-N1-SOX5-WT* vector and those transfected with the *pEGFP-N1-SOX5-MUT* vector. Specifically, the mRNA levels of the mutant *SOX5* were elevated in comparison to the wild-type in 293T cells and Neuro-2a cells (Fig. 2B). Furthermore, the levels of the *SOX5* p.Thr74Met variant protein and its protein stability were enhanced relative to the wild-type protein in Neuro-2a cells and 293T cells (Fig. 2C–D, F–K). In addition, transfection with mutant *SOX5* resulted in a reduction of cyclin A protein levels compared to transfection with wild-type *SOX5* in Neuro-2a cells (Fig. 2C, E). However, the subcellular localization of the mutant *SOX5* was observed to be identical to that of the wild-type, with both being localized in the nucleus in 293T cells and Neuro-2a cells (Fig. 3A). Lastly, the cell cycle analysis demonstrated that the proportion of cells in the S phase was lower following transfection with mutant *SOX5* compared to transfection with the wild-type in Neuro-2a cells (Fig. 3B–C). The CCK-8 assay demonstrated that the proliferation rate of cells harboring the *SOX5* p.Thr74Met variant was reduced at the 36-h mark (Fig. 3D). Additionally, we observed a decreased proliferation rate in Neuro-2a cells co-expressing wild-type and mutant proteins at the same time point (Fig. 3D). To further investigate, we evaluated the expression levels of the *ACAN*, *AXIN2*, *SOX9* and *PDGFRA* genes in 293T cells overexpressing *SOX5*. Following the overexpression of the wild-type and mutant *SOX5* genes, a reduction in mRNA levels of these four genes was observed in cells expressing the mutant *SOX5* compared to those expressing the wild-type (Fig. 3E–H).

## Discussion

In a Chinese family presenting with intellectual disability, we identified a missense variant (c.221C > T, p.Thr74Met) in the *SOX5* gene. The proband exhibited the ability to walk by the age of 3 years, experienced a delay in speech development by the age of 4 years, achieved a score of 52 on an intelligence assessment, and was diagnosed with intellectual developmental delay. Her father exhibited similar clinical manifestations. Sequencing analysis identified a heterozygous variation in the *SOX11* gene, c.674A > G (p.Asp225Gly), and a heterozygous variation in the *SOX5* gene, c.221C > T (p.Thr74Met). Previous studies have reported *SOX5* as the pathogenic gene associated with Lamb-Shaffer syndrome, characterized by mild to moderate intellectual disability, deficits in language and motor skills, behavior deficits including autistic traits, hypotonia, short stature, and subtle distinctive facial features [16–18]. What’s more, the *SOX11* variant is implicated in Coffin-Siris syndrome, a condition traditionally marked by developmental or cognitive delays, phalangeal hypoplasia, distinctive facial features, hypotonia, autistic traits, hirsutism/hypertrichosis, sparse scalp hair and various systemic abnormalities affecting the cardiac, gastrointestinal and urinary systems [19, 20]. Differentiating Lamb-Shaffer syndrome from Coffin-Siris syndrome based on clinical symptoms alone presents significant challenges. In the family under study, the phenotype is comparatively mild, manifesting as mild intellectual disability, along with language and motor deficits. In this family, *SOX11* variant (c.674A > G, p.Asp225Gly) is present in the proband's unaffected brother (II:1). According to the genotype and phenotype co-segregation model, *SOX5* variant (c.221C > T, p.Thr74Met) is identified as the pathogenic gene, whereas *SOX11* is not considered pathogenic gene. Previous research has documented that, despite a positive family history of late-onset Alzheimer's disease (LOAD), the *SOX5* variant (c.221C > T, p.Thr74Met) was detected in healthy individuals, and this variant was thus interpreted as being protective [21]. However, the exact mechanism underlying this protective effect remains unknown. Based on our comprehensive analysis utilizing high precision clinical exon sequencing, Sanger sequencing, and bioinformatics analysis, we predict that this *SOX5* variant (c.221C > T, p.Thr74Met) is harmful and pathogenic, leading to structural disruption of the *SOX5* protein. (Table 2 and Fig. 1C–D). In addition, because this *SOX5* variant Thr74Met has also been found in healthy individuals from the LOAD family, we speculate that it may exhibit incomplete penetrance.

The *SOX5* variant (c.221C > T, p.Thr74Met) is located within the first Coiled-Coil domain (amino acids 193–274), which serves as molecular spacers separating functional domains or large macromolecular complexes and potentially facilitates protein–protein interactions [14]. According to the predictive software I-TASSER, the HMG domain of the *SOX5* p.Thr74Met variant protein exhibits notable structural alterations, with certain β-sheet regions transforming into helical configurations, as illustrated within the red box in Fig. 1D. The HMG domain serves as the interaction interface between *SOX5* and *SOX9*, and it also functions as the DNA binding region [22]. Taken together, these variants induce modifications in the protein structure, potentially influencing its interactions with other proteins, such as *SOX9*.

As a transcription factor, *SOX5* plays a significant role in the development of the nervous system. The development of the central nervous system can be promoted by appropriate levels of *SOX5* protein, with its overexpression aiding in the repair of apoptosis induced by lipopolysaccharides [5]. However, premature elevation of *SOX5* levels may result in decreased cell proliferation and increased apoptosis, consequently reducing the total number of neuroepithelial cells in HH14–16 embryos [23]. Therefore, the down-regulation of *SOX5* is necessary for neuronal differentiation. In addition, overexpression of L-*SOX5* can inhibit neurite outgrowth in primary hippocampal pyramidal neurons, which affects neuronal differentiation [24]. In our study, the stability of *SOX5* mutant protein (p.Thr74Met) was higher than that of the wild-type protein, as were the corresponding mRNA and protein levels (Fig. 2B–D, F–K). The enhanced stability of *SOX5* mutant protein may account for the observed increase in protein levels. We speculate that the increased stability of the *SOX5* mutant protein (c.221C > T, p.Thr74Met) could potentially influence neuronal differentiation, leading to diminished activation of cell proliferation and apoptosis, as well as reduced protrusion growth in primary hippocampal pyramidal neurons.

The *SOX5* protein plays a crucial role in regulating the cell cycle, proliferation, apoptosis and differentiation [25]. In adipocytes, knockdown of *SOX5* enhances cell proliferation by upregulating gene expression during the cell cycle, decreasing the G1 phase cell population and increasing the populations in the S and G2 phases [26]. Conversely, overexpression of *SOX5* in neural progenitors prompts cell cycle exit, resulting in cell accumulation in G0 phase or prolonged retention in G1 phase [23]. Our study demonstrated that mutant *SOX5* (c.221C > T, p.Thr74Met) led to a reduction in the S phase cell population, while no significant differences were observed in the G1 and G2 phases when compared to the wild-type (Fig. 3B–C). This variant resulted in decreased expression of cyclin A in *SOX5* variants relative to the wild-type (Fig. 2C, E). Cyclin A is known to regulate kinases during the S phase and the S/G2 phase transition [27], while Cyclin A/cdk1 kinase guides mitosis [28]. Additionally, the *SOX5* p.Thr74Met variant was found to slow the proliferation of Neuro-2a cells, and co-transfection with both the wild-type and mutant forms also resulted in inhibited proliferation. Consequently, we hypothesize that the *SOX5* variant (c.221C > T, p.Thr74Met) impedes cell cycle progression, induces premature cell cycle exit, and suppresses cellular proliferation, thereby influencing neuronal differentiation. This finding is particularly noteworthy in the context of Alzheimer's disease (AD), where cell cycle reactivation is a characteristic feature of neurodegenerative process [29]. We propose that the *SOX5* p.Thr74Met variant's ability to inhibit cell cycle progression may prevent such reactivation, potentially conferring protective effects against AD. Nevertheless, the specific mechanisms responsible for this dual effect warrant further investigation and exploration.

We also examined the effects of *SOX5*-induced alterations on the regulation of gene expression for *ACAN*, *AXIN2*, *SOX9* and *PDGFRA* (Fig. 3E–H). We speculate that the increased stability and expression of *SOX5* lead to the downregulation of its target gene expression, thereby contributing to disease onset. When co-activated by SOXH and the SOX Trio (*SOX5*, *SOX6* and *SOX9*), aggrecan (*ACAN*) plays a key role in cartilage and bone morphogenesis by regulating essential growth factors and signaling molecules [30, 31]. It has been reported that a variant of *ACAN* (c.4634delT, Leu1545Profs*11) is associated with short stature and intervertebral disc disease [32]. *ACAN*, which may play a central role in the regulation of adult brain plasticity, is an important component in the assembly and maintenance of the structure and function of perineuronal nets in the adult brain [33]. Therefore, *ACAN* serves as a key regulator in bone and neuronal development. In our study, we observed a reduction in *ACAN* mRNA levels in mutant *SOX5* (c.221C > T, p.Thr74Met) cells compared to wild-type. The decline of *ACAN* expression may impact density of GABAergic synapses [34] and the metabolic processes of chondrocytes [35]. Furthermore, a separate study demonstrated that the overexpression of *SOX5* can influence *AXIN2* expression [36]. The mutant *SOX5* (p.Thr74Met) cells exhibited a reduction in *AXIN2* mRNA levels compared to the wild-type cells. The *AXIN2* protein is a transcriptional target of active WNT signaling and participates in the regulation of the WNT pathway across various tissues and systems [37]. Thus, abnormal expression of *AXIN2* may affect the WNT signaling pathway, which is crucial for bone and neural development. Alongside *SOX5* and *SOX6*, the proteins *SOX9* and *SOX10* are involved in regulating oligodendrocyte differentiation, migration and survival by activating *PDGFRA* expression in oligodendrocyte progenitor cells [10]. The *PDGFRA* protein is capable of activating the ERK signaling pathway and regulating the migration of oligodendrocyte progenitor cells [38]. In summary, *SOX5* collaborates with *SOX9* to promote the expression of *ACAN* and *PDGFRA*, and it further augments the transcription of *AXIN2* [10, 23, 39]. However, the *SOX5* p.Thr74Met variant protein exhibits inhibitory effects on the expression of *ACAN*, *PDGFRA*, and *AXIN2*, while also leading to a downregulation of *SOX9* expression. Considering that the HMG domain serves as the interaction region between *SOX5* and *SOX9* and the DNA-binding region, we speculate that the p.Thr74Met variant induces alterations in the HMG domain of *SOX5*, potentially affecting its interactions with *SOX9*. This may impair its function to activate the expression of target genes, thereby exerting an inhibitory effect. In our study, the *SOX5* variant (c.221C > T, p.Thr74Met) was observed to downregulate the expression of its target genes (*ACAN*, *AXIN2*, *SOX9* and *PDGFRA*), which could influence the development and differentiation of nerves and bones.

## Conclusion

The *SOX5* variant (c.221C > T, p.Thr74Met) was found to enhance protein expression and stability, subsequently downregulating the expression of *SOX5* target genes, including *ACAN*, *AXIN2*, *SOX9* and *PDGFRA*, through a dominant negative effect, ultimately leading to cell cycle inhibition. The pathogenicity of the *SOX5* c.221C > T variant was confirmed in a second-generation Chinese family. Knowledge of the underlying mechanisms may offer valuable insights for the development of novel therapeutic strategies for patients with intellectual disabilities associated with *SOX5* variants.

## Data Availability

The datasets generated and/or analysed during the current study are not publicly available due to ethics or patient privacy but are available from the corresponding author on reasonable request.

## Abbreviations

HMG: High mobility group
3D: Three-dimensional
HEK: Human embryonic kidney
qPCR: Quantitative Real-time PCR
GFP: Green fluorescent protein
LOAD: Late-onset Alzheimer disease
